# Clinical Features and Cutaneous Manifestations of Juvenile and Adult Patients of Dermatomyositis Associated with Myositis-Specific Autoantibodies

**DOI:** 10.3390/jcm10081725

**Published:** 2021-04-16

**Authors:** Naoko Okiyama

**Affiliations:** Department of Dermatology, Faculty of Medicine, University of Tsukuba, Ibaraki 305-8575, Japan; naoko.okiyama@md.tsukuba.ac.jp; Tel.: +81-29-853-3128

**Keywords:** dermatomyositis, skin, autoantibody

## Abstract

Dermatomyositis is one of the idiopathic inflammatory myopathies, which is characterized with specific skin manifestations, and considered as an autoimmune disease. Dermatomyositis is a heterogeneous disorder with various presences, severities and characteristics of myositis, dermatitis, and interstitial lung disease. Our and others’ data showed that myositis-specific autoantibodies have been associated with distinct clinical features. This article reviewed the epidemiology and characteristic clinical features of the different types of antibody-associated dermatomyositis in adult and juvenile patients, which include the severity of myopathy, the potential complication of interstitial lung disease, potential association with malignancies, and characteristic cutaneous manifestations.

## 1. Introduction

Dermatomyositis (DM) is one of idiopathic inflammatory myopathies (IIMs), which is characterized with specific skin manifestations, the pathologies of which are considered autoimmune diseases. DM is a heterogeneous disorder that can occur in adults and juveniles, and it has various phenotypes, including myositis, dermatitis, and interstitial lung disease (ILD) [1]. Recently, several myositis-specific autoantibodies not detected in patients with inherited muscle diseases have been identified [2], and they include anti-transcriptional intermediary factor 1 (TIF1) γ, anti-nuclear matrix protein 2 (NXP2), anti-melanoma differentiation-associated protein 5 (MDA5), anti-Mi-2, and anti-small ubiquitin-like modifier activating enzyme (SAE) antibodies, in addition to already established anti-aminoacyl-transfer RNA synthetase (ARS) antibodies, including anti-Jo-1 antibody. These autoantibodies are not only highly disease-specific; our and others’ data showed that they are associated with distinct clinical features [3,4]. In other words, these myositis-specific autoantibodies are useful to define DM better than before. This article reviewed the epidemiology and characteristic clinical features of the different types of antibody-associated DM in adult and juvenile patients, which include the severity of myopathy, the potential complication of ILD, and potential association with malignancies. The characteristic cutaneous manifestations are described in a separate chapter.

## 2. Epidemiology and Clinical Features of Subgroups Classified According to Myositis-Specific Autoantibodies

The characteristics of each subgroup are detailed in the following chapters, and summarized in Table 1.

### 2.1. Anti-TIF1 Antibody-Positive DM

The anti-TIF1 antibodies were originally reported as anti-155/140 and anti-p155 antibodies [5,6]. The antibodies target a 155-kDa nuclear protein, sometimes with a 140-kDa protein, which were subsequently identified as TIF1 family proteins belonging to the tripartite motif (TRIM) superfamily: TIF1γ (TRIM33) and TIF1α (TRIM24), respectively. A number of reports from the USA, Europe and Japan revealed that the anti-TIF1γ antibody is associated with malignancies, especially in older adult patients [7,8,9]. In contrast, 35% of juvenile DM (JDM) patients have anti-TIF1γ antibodies by a review article depending on several large registries developed in the USA, Canada and the UK [10], while 17.4% and 22% of JDM patients do in a report of 58 cases from Turkey and another report of 64 cases from Argentina [11,12]. Moreover, the patients do not develop malignancies [6,10,11]. A chronic illness course and lipodystrophy have been associated with anti-TIF1 antibodies in JDM patients [10], and one-third of anti-TIF1 antibody-positive JDM patients have a relapse [11].

### 2.2. Anti-NXP2 Antibody-Positive DM

The anti-nuclear matrix protein 2 (NXP2) antibody was first identified in a cohort of JDM/juvenile polymyositis (JPM) patients, and was originally termed an anti-MJ antibody [12]. The cohort studies including a review of several large registries, and two cohorts with small case numbers, detected anti-NXP2 antibodies in 22%–25% of patients with JDM [10,11,12]. A cohort study reported that severe myopathy characterized by muscle contractures and atrophy was associated with anti-NXP2 antibody-positive JDM [12], and 43% of anti-NXP2 antibody-positive JDM patients have a relapse [11]. The risk of ILD was suggested in anti-NXP2 antibody-positive JDM, as well as anti-MDA5 antibody-positive patients [11]. Two cohort studies on adult, but not juvenile, PM/DM patients in Japan and the US suggested an association between the anti-NXP2 antibody and malignancy [8,13]. We also found that adult patients of anti-NXP2 antibody-positive IIMs in our Japanse cohort had a higher prevalence of malignancy than the general population with an increased age-standardized incidence ratio of malignancies (unpublished data).

### 2.3. Anti-MDA5 Antibody-Positive DM

The anti-MDA5 antibody, which was termed an anti-CADM-140 antibody that reacts with a 140-kDa cytoplasmic protein [14], has been reported to present a high specificity for clinically amyopathic DM (CADM) accompanied by rapidly progressive ILD (RP-ILD) [15]. The target antigen of anti-MDA5 antibody was subsequently identified as the retinoic acid-inducible gene I-like receptor MDA5/IFIH1 (interferon induced with helicase C domain protein 1). The anti-MDA5 antibody is frequently detected among DM patients in Asia (36.6% (53/145 cases) in China, and 15.8% (26/165 cases) in Japan) [16] and at a low frequency (2.8% (21/748 cases)) in a cohort of DM patients in a combined European cohort in which 87.4% of enrolled patients were Caucasian [17]. In JDM patients, anti-MDA5 antibodies were detected in 28%–33% of a Japanese cohort [18,19] and 7.4% of a UK-based cohort [20]. The disparity might be dependent on the ethic difference, environmental factors, or low sensitivity due to the absence of myopathies in the patients of anti-MDA5 antibody-positive DM.

Some case series have reported CADM frequencies of approximately 40% in anti-MDA5 antibody-positive DM adult patients; in the juvenile cohort, it was reported that anti-MDA5 antibody-positive patients had a lower prevalence of severe muscle weakness than anti-MDA5 antibody-negative JDM patients [20]. A meta-analysis of 16 studies estimated that the pooled sensitivity and specificity of anti-MDA5 antibodies for RP-ILD were 77% (95% confidence interval (CI), 64%–87%) and 86% (95% CI, 79%–90%), respectively, with a pooled diagnostic odds ratio of 20.41 (95% CI, 9.02–46.20) [16]. The anti-MDA5 antibody has been reported as a diagnostic and predictive marker for ILD, especially RP-ILD, even in JDM patients [19,21]. Nevertheless, only eight of the 44 Japanese juvenile patients (18%) and none of the UK juvenile patients developed RP-ILD [19,20].

### 2.4. Anti-Mi-2 Antibody-Positive DM

The anti-Mi-2 antibody mainly reacts to Mi-2β, a component of the nucleosome-remodeling deacetylase complex. The anti-Mi-2 antibody was detected less frequently in JDM patients (3–8.7%) [10,11] compared to adult DM patients (12%) [17]. Patients with the anti-Mi-2 antibody tend to present with severe myositis [22,23]; however, they typically respond well to therapy [24,25] and show clinical remission [26].

### 2.5. Anti-SAE Antibody-Positive DM

The anti-SAE antibody, which targets a heterodimer of SAE1 (40 kDa) and SAE2 (90 kDa), was identified in DM patients in 2007 [27]. This antibody was observed in approximately 6% of patients with DM [17], and it was associated with inflammatory myopathy, characterized by extensive rash and dysphagia [27,28]. While anti-SAE antibody-positive juvenile patients have rarely been reported, we reported a case complicated with ILD [29].

### 2.6. Anti-ARS Antibody-Positive DM

Anti-ARS antibodies include anti-Jo-1, anti-PL-7, anti-PL-12, anti-EJ, anti-OJ, anti-KS, anti-Ha, and anti-Zo. Patients with a positivity of thease antibodies share characteristic clinical symptoms such as myositis, ILD, arthritis/arthralgia, Raynaud’s phenomenon, and fever, moreover, are distinguished from DM and PM. Thus, the term “antisynthetase syndrome” is also used to describe this population [30]. A small population of anti-ARS antibody-positive patients has been reported in JDM/JPM [11,18].

**Table 1:** Subgroups of juvenile dermatomyositis characterized by myositis-specific autoantibodies.

Antibody	TIF1	NXP2	MDA5	Mi-2	SAE	ARS
Population	17–35% [10,11,12]	22–25% [10,11,12]	28–33% (Japan) [18,19] 7.4% (UK) [20]	3–8.7% [10,11]	Rare	Rare [11,18]
Myopathy	Severe, persistent [10], relapse [11]	Severe, persistent [12], relapse [11]	None–mild [20]	Severe [22,23], responsive to therapy [24,25]	―	―
Interstitial lung disease	Improbable	Frequent [11]	Frequent, rapidly progress [19,21]	Probable	―	Frequent, chronic [30]

## 3. Characteristic Cutaneous Manifestations

The characteristic cutaneous manifestations of each subgroup are detailed in the following chapters and summarized in Table 2.

### 3.1. Anti-TIF1 Antibody-Positive DM

Patients with anti-TIF1γ antibody-associated DM were reported to show severe cutaneous manifestations, including the V-neck sign, shawl sign, heliotrope rash, Gottron’s papules/sign, and flagellate erythema [5,6]. These rashes are associated with exposure to ultraviolet light. Exposere to ultraviolet light increased the odds of the anti-TIF1 antibody being present in JDM patients [31]. Erythroderma, which is reported in 15% of JDM patients, is more common in those with the anti-TIF1 antibody [32], and these characteristic cutaneous manifestations were known as palmar hyperkeratotic papules, psoriasis-like lesions, and hypopigmented and “red on white” telangiectatic patches in a previous report [33]. Half of 10 patients with anti-TIF1 antibody-positive JDM in a cohort of Turkish JDM patients were reported to develop calcinosis [11].

### 3.2. Anti-NXP2 Antibody-Positive DM

This antibody was first detected in JDM patients; however, it can be detected in JPM patients [10]. We reported that DM sine dermatitis was associated with the anti-NXP2 antibody [34] and found that a part of juvenile patients and half of adult patients presented with the PM phenotype without DM-specific cutaneous manifestations in our anti-NXP2 antibody-positive case series (unpublished data). The anti-NXP2 antibody-positive DM may be characterized by the scantiness of DM-specific cutaneous manifestations.

In contrast, both juvenile and adult myopathy patients positive for anti-NXP2 antibodies have a high risk of calcinosis [35,36]. FDG-PET detected calcinosis [37]. However, in a study, the anti-MDA5 and anti-TIF1, as well as anti-NXP2 antibodies were associated with calcinosis in a cohort of JDM patients [11], and JDM onset within 6 years of age was a predictive factor for the development of calcinosis [11]. As calcinosis is not specific to anti-NXP2 antibody-positive DM, but for vascular damage, various risk factors, which include local pressure due to muscle weakness and increased blood viscosity accompanied by inflammation, for the development of calcinosis exist.

### 3.3. Anti-MDA5 Antibody-Positive DM

Cutaneous ulceration due to vascular injuries was reported to be related to rapidly progressive ILD in patients of anti-MDA5 antibody-associated DM [38,39]. Caracteristic palmar violaceous macules/papules [38,40] were often observed, in which vasculopathy in the medium and small dermal vessels was frequently observed [38].

We previously performed analyses on the histological findings of finger lesions from 74 DM patients characterized by myositis-specific autoantibodies (anti-MDA5, anti-TIF1γ, and anti-ARS) [41]. The analyses also showed that vascular injury in the upper dermis was frequently observed in anti-MDA5 antibody-positive DM [41]. Immunohistochemistry for myxovirus resistance A (MxA), one of type I interferon activity-associated molecules, revealed that epidermal keratinocytes express MxA in the skin samples of anti-MDA5 antibody-positive DM patients than in anti-TIF1γ antibody-positive DM patients, while few keratinocytes were expressed in anti-ARS antibody-positive DM patients [41]. Anti-MDA5 antibody-positive DM and JDM, but not anti-synthetase syndrome, are known as interferonopathies mediated by type I interferons based on our data and previous studies on muscle and blood samples [42,43,44,45].

### 3.4. Anti-Mi-2 Antibody-Positive DM

Few characteristic cutaneous manifestations have been reported for the anti-Mi-2 antibody DM. A previous retrospective study of 64 cases revealed that anti-Mi-2 antibody DM patients tended to present with a classic DM rash (Gottron papules/sign, heliotrope rash, periungual erythema, and/or violaceous rach including Holster sign) without additional skin changes, such as calcinosis, ulcers, panniculitis, and/or mechanic’s hands [46]. Anti-Mi-2 antibody-positive DM is named “classic DM”. Unlike in adults, V- and shawl-sign rashes and cuticular overgrowth are not associated with anti-Mi-2 antibody-positive JDM [32].

### 3.5. Anti-SAE Antibody-Positive DM

Patients with anti-SAE antibody-positive DM usually demonstrate extensive rashes [27], and we termed the cutaneous manifestations as erythroderma with the “angel wings” sign, which means diffuse erythema sparing the parts on the scapulas [28]. Although very few anti-SAE antibody-positive JDM patients were presented, we encountered a case of anti-SAE antibody-positive JDM who presented with extensive facial erythema without erythroderma [29].

### 3.6. Anti-ARS Antibody-Positive DM

Mechanic’s hands are characterized by hyperkeratotic erythema on the sides of the thumbs and forefingers [47]. Patients with antisynthetase syndrome, including those with anti-ARS antibody-associated DM, generally present the mechanic’s hands [30]. It is sometimes difficult for dermatologists to distinguish the mechanic’s hands and Gottron’s papules/sign of anti-ARS antibody-positive DM patients from eczema and/or psoriasis. Our previous analysis of the histological findings of finger lesions from 74 DM patients characterized according to myositis-specific autoantibodies (anti-MDA5, anti-TIF1γ, and anti-ARS) indicated that eczematous reactions (spongiosis) and psoriasiform dermatitis (psoriasiform acanthosis and parakeratosis) were significantly more frequently observed in skin samples of anti-ARS antibody-positive patients than in anti-MDA5 or TIFγ antibody-positive patients [41]. In addition, MxA expression was rarely observed in skin samples [41], as well as muscle samples [42,43], of anti-ARS antibody-positive patients.

In JDM patients, anti-ARS antibody is also associated with mechanic’s hands, Raynaud’s phenomenon, and ILD [32].

**Table 2:** Cutaneous manifestations characterized by myositis-specific autoantibodies.

Antibody	TIF1	NXP2	MDA5	Mi-2	SAE	ARS
Classic (Gottron papules/sign, heliotrope rash)	Frequent [5,6]	Sometimes absent [10]	Frequent, violaceous [38]	Frequent [46]	Frequent [27]	Sometimes absent [30], accompanied by eczematous/psoriasiform reactions [41]
Additional	Photosensitivity [31], erythroderma [32], calcinosis [11]	Calcinosis [35,36]	Palmar violaceous macules/papules and skin ulceration associated with vasculopathy [38,40,41]	Rare [46]	Erythroderma, Angel wings sign [28]	Mechanic’s hands, Raynaud’s phenomenon [30]

## 4. Conclusions

DM and JDM include a broad spectrum, and may include cases of PM phenotypes without DM-specific cutaneous manifestations, Gottron papules/sign and heliotrope rash. Moreover, the severity of myopathy, the potential complication of ILD, and potential association with malignancies also present a broad spectrum, not only between juveniles and adults, but also among subgroups besed on these myositis-specific autoantibodies. Further studies, and an international and a wide range of investigators’ consensus, are needed to classify adult and juvenile DM/PM cases to subgroups based on the myositis-specific autoantibodies. In addition, murine models for some of these subgrops, the anti-ARS antibody-associated group [48] and the anti-TIF1 antibody-associated group [49], have been established. These murine models might be useful to provide a basis for the development of subgroup-specific DM therapies.

## References

[1] Lilleker J.B., Vencovsky J., Wang G., Wedderburn L.R., Diederichsen L.P., Schmidt J., Oakley P., Benveniste O., Danieli M.G., Danko K. (2018). The EuroMyositis registry: An international collaborative tool to facilitate myositis research. Ann. Rheum. Dis..

[2] Mammen A.L., Casciola-Rosen L., Christopher-Stine L., Lloyd T.E., Wagner K.R. (2015). Myositis-specific autoantibodies are specific for myositis compared to genetic muscle disease: Table. Neurol. Neuroimmunol. Neuroinflamm..

[3] Tansley S.L., McHugh N.J., Wedderburn L.R. (2013). Adult and juvenile dermatomyositis: Are the distinct clinical features explained by our current understanding of serological subgroups and pathogenic mechanisms?. Arthritis Res. Ther..

[4] Lundberg I.E., De Visser M., Werth V.P. (2018). Classification of myositis. Nat. Rev. Rheumatol..

[5] Kaji K., Fujimoto M., Hasegawa M., Kondo M., Saito Y., Komura K., Matsushita T., Orito H., Hamaguchi Y., Yanaba K. (2006). Identification of a novel autoantibody reactive with 155 and 140 kDa nuclear proteins in patients with dermatomyositis: An association with malignancy. Rheumatology.

[6] Targoff I.N., Mamyrova G., Trieu E.P., Perurena O., Koneru B., O’Hanlon T.P., Miller F.W., Rider L.G. (2006). Childhood Myositis Heterogeneity and International Myositis Collaborative Study Groups A novel autoantibody to a 155-kd protein is associated with dermatomyositis. Arthritis Rheum..

[7] Fujimoto M., Hamaguchi Y., Kaji K., Matsushita T., Ichimura Y., Kodera M., Ishiguro N., Ueda-Hayakawa I., Asano Y., Ogawa F. (2012). Myositis-specific anti-155/140 autoantibodies target transcription intermediary factor 1 family proteins. Arthritis Rheum..

[8] Fiorentino D.F., Chung L.S., Christopher-Stine L., Zaba L., Li S., Mammen A.L., Rosen A., Casciola-Rosen L. (2013). Most patients with cancer-associated dermatomyositis have antibodies to nuclear matrix protein NXP-2 or transcription intermediary factor 1gamma. Arthritis Rheum..

[9] Venalis P., Selickaja S., Lundberg K., Rugiene R., Lundberg I.E. (2018). Association of Anti-Transcription Intermediary Factor 1gamma Antibodies With Paraneoplastic Rheumatic Syndromes Other Than Dermatomyositis. Arthritis Care Res..

[10] Rider L.G., Nistala K. (2016). The juvenile idiopathic inflammatory myopathies: Pathogenesis, clinical and autoantibody phenotypes, and outcomes. J. Intern. Med..

[11] Sag E., Demir S., Bilginer Y., Talim B., Haliloglu G., Topaloglu H., Ozen S. (2021). Clinical features, muscle biopsy scores, myositis specific antibody profiles and outcome in juvenile dermatomyositis. Semin. Arthritis Rheum..

[12] Espada G., Cocco J.A.M., Fertig N., Oddis C.V. (2009). Clinical and Serologic Characterization of an Argentine Pediatric Myositis Cohort: Identification of a Novel Autoantibody (anti-MJ) to a 142-kDa Protein. J. Rheumatol..

[13] Ichimura Y., Matsushita T., Hamaguchi Y., Kaji K., Hasegawa M., Tanino Y., Inokoshi Y., Kawai K., Kanekura T., Habuchi M. (2012). Anti-NXP2 autoantibodies in adult patients with idiopathic inflammatory myopathies: Possible association with malignancy. Ann. Rheum. Dis..

[14] Sato S., Hirakata M., Kuwana M., Suwa A., Inada S., Mimori T., Nishikawa T., Oddis C.V., Ikeda Y. (2005). Autoantibodies to a 140-kd polypeptide, CADM-140, in Japanese patients with clinically amyopathic dermatomyositis. Arthritis Rheum..

[15] Fujimoto M., Watanabe R., Ishitsuka Y., Okiyama N. (2016). Recent advances in dermatomyositis-specific autoantibodies. Curr. Opin. Rheumatol..

[16] Chen Z., Cao M., Plana M.N., Liang J., Cai H., Kuwana M., Sun L. (2013). Utility of Anti-Melanoma Differentiation-Associated Gene 5 Antibody Measurement in Identifying Patients With Dermatomyositis and a High Risk for Developing Rapidly Progressive Interstitial Lung Disease: A Review of the Literature and a Meta-Analysis. Arthritis Rheum..

[17] Betteridge Z., Tansley S., Shaddick G., Chinoy H., Cooper R., New R., Lilleker J., Vencovsky J., Chazarain L., Danko K. (2019). Frequency, mutual exclusivity and clinical associations of myositis autoantibodies in a combined European cohort of idiopathic inflammatory myopathy patients. J. Autoimmun..

[18] Ueki M., Kobayashi I., Takezaki S., Tozawa Y., Okura Y., Yamada M., Kuwana M., Ariga T. (2019). Myositis-specific autoantibodies in Japanese patients with juvenile idiopathic inflammatory myopathies. Mod. Rheumatol..

[19] Kobayashi N., Takezaki S., Kobayashi I., Iwata N., Mori M., Nagai K., Nakano N., Miyoshi M., Kinjo N., Murata T. (2015). Clinical and laboratory features of fatal rapidly progressive interstitial lung disease associated with juvenile dermatomyositis. Rheumatology.

[20] Tansley S.L., Betteridge Z.E., Gunawardena H., Jacques T.S., Owens C.M., Pilkington C., Arnold K., Yasin S., Moraitis E., Wedderburn L.R. (2014). Anti-MDA5 autoantibodies in juvenile dermatomyositis identify a distinct clinical phenotype: A prospective cohort study. Arthritis Res. Ther..

[21] Kobayashi I., Okura Y., Yamada M., Kawamura N., Kuwana M., Ariga T. (2011). Anti-Melanoma Differentiation-Associated Gene 5 Antibody is a Diagnostic and Predictive Marker for Interstitial Lung Diseases Associated with Juvenile Dermatomyositis. J. Pediatrics.

[22] Pinal-Fernandez I., Mecoli C.A., Casal-Dominguez M., Pak K., Hosono Y., Huapaya J., Huang W., Albayda J., Tiniakou E., Paik J.J. (2019). More prominent muscle involvement in patients with dermatomyositis with anti-Mi2 autoantibodies. Neurology.

[23] Tanboon J., Inoue M., Hirakawa S., Tachimori H., Hayashi S., Noguchi S., Suzuki S., Okiyama N., Fujimoto M., Nishino I. (2021). Pathologic Features of Anti-Mi-2 Dermatomyositis. Neurology.

[24] Hamaguchi Y., Kuwana M., Hoshino K., Hasegawa M., Kaji K., Matsushita T., Komura K., Nakamura M., Kodera M., Suga N. (2011). Clinical correlations with dermatomyositis-specific autoantibodies in adult Japanese patients with dermatomyositis: A multicenter cross-sectional study. Arch. Dermatol..

[25] Hengstman G.J., Vree Egberts W.T., Seelig H.P., Lundberg I.E., Moutsopoulos H.M., Doria A., Mosca M., Vencovsky J., van Venrooji W.J., van Engelen B.G.M. (2006). Clinical characteristics of patients with myositis and autoantibodies to different fragments of the Mi-2 beta antigen. Ann. Rheum. Dis..

[26] Liang L., Zhang Y.-M., Chen H., Ye L.-F., Li S.-S., Lu X., Wang G.-C., Peng Q.-L. (2020). Anti-Mi-2 antibodies characterize a distinct clinical subset of dermatomyositis with favourable prognosis. Eur. J. Dermatol..

[27] Betteridge Z., Gunawardena H., North J., Slinn J., McHugh N.J. (2007). Identification of a novel autoantibody directed against small ubiquitin-like modifier activating enzyme in dermatomyositis. Arthritis Rheum..

[28] Inoue S., Okiyama N., Shobo M., Motegi S.-I., Hirano H., Nakagawa Y., Saito A., Nakamura Y., Ishitsuka Y., Fujisawa Y. (2018). Diffuse erythema with ‘angel wings’ sign in Japanese patients with anti-small ubiquitin-like modifier activating enzyme antibody-associated dermatomyositis. Br. J. Dermatol..

[29] Kishi T., Tani Y., Okiyama N., Mizuochi K., Ichimura Y., Harigai M., Nagata S., Miyamae T. (2021). Anti-SAE autoantibody-positive Japanese patient with juvenile dermatomyositis complicated with interstitial lung disease—A case report. Pediatric Rheumatol..

[30] Lega J.-C., Fabien N., Reynaud Q., Durieu I., Durupt S., Dutertre M., Cordier J.-F., Cottin V. (2014). The clinical phenotype associated with myositis-specific and associated autoantibodies: A meta-analysis revisiting the so-called antisynthetase syndrome. Autoimmun. Rev..

[31] Shah M., Targoff I.N., Rice M.M., Miller F.W., Rider L.G. (2013). With the Childhood Myositis Heterogeneity Collaborative Study Group Brief Report: Ultraviolet Radiation Exposure Is Associated With Clinical and Autoantibody Phenotypes in Juvenile Myositis. Arthritis Rheum..

[32] Rider L.G., Shah M., Mamyrova G., Huber A.M., Rice M.M., Targoff I.N., Miller F.W. (2013). The Myositis Autoantibody Phenotypes of the Juvenile Idiopathic Inflammatory Myopathies. Medicine.

[33] Fiorentino D.F., Kuo K., Chung L., Zaba L., Li S., Casciola-Rosen L. (2015). Distinctive cutaneous and systemic features associated with antitranscriptional intermediary factor-1gamma antibodies in adults with dermatomyositis. J. Am. Acad. Dermatol..

[34] Inoue M., Tanboon J., Hirakawa S., Komaki H., Fukushima T., Awano H., Tajima T., Yamazaki K., Hayashi R., Mori T. (2020). Association of Dermatomyositis Sine Dermatitis With Anti–Nuclear Matrix Protein 2 Autoantibodies. JAMA Neurol..

[35] Zhong L., Yu Z., Song H. (2019). Association of anti-nuclear matrix protein 2 antibody with complications in patients with idiopathic inflammatory myopathies: A meta-analysis of 20 cohorts. Clin. Immunol..

[36] Tansley S.L., Betteridge Z.E., Shaddick G., Gunawardena H., Arnold K., Wedderburn L.R., McHugh N.J. (2014). Calcinosis in juvenile dermatomyositis is influenced by both anti-NXP2 autoantibody status and age at disease onset. Rheumatology.

[37] Nakamura N., Izumi R., Hoshi Y., Takai Y., Ono R., Suzuki N., Nagai T., Ishii Y., Ishii T., Harigae H. (2019). FDG-PET detects extensive calcinosis cutis in anti-NXP2 antibody-positive dermatomyositis. Rheumatology.

[38] Fiorentino D., Chung L., Zwerner J., Rosen A., Casciola-Rosen L. (2011). The mucocutaneous and systemic phenotype of dermatomyositis patients with antibodies to MDA5 (CADM-140): A retrospective study. J. Am. Acad. Dermatol..

[39] Narang N.S., Casciola-Rosen L., Li S., Chung L., Fiorentino D.F. (2015). Cutaneous Ulceration in Dermatomyositis: Association With Anti-Melanoma Differentiation-Associated Gene 5 Antibodies and Interstitial Lung Disease. Arthritis Rheum..

[40] Koguchi-Yoshioka H., Okiyama N., Iwamoto K., Matsumura Y., Ogawa T., Inoue S., Watanabe R., Fujimoto M. (2017). Intravenous immunoglobulin contributes to the control of antimelanoma differentiation-associated protein 5 antibody-associated dermatomyositis with palmar violaceous macules/papules. Br. J. Dermatol..

[41] Okiyama N., Yamaguchi Y., Kodera M., Hamaguchi Y., Yokozeki H., Ishiguro N., Fujimoto M. (2019). Distinct Histopathologic Patterns of Finger Eruptions in Dermatomyositis Based on Myositis-Specific Autoantibody Profiles. JAMA Dermatol..

[42] Noguchi E., Uruha A., Suzuki S., Hamanaka K., Ohnuki Y., Tsugawa J., Watanabe Y., Nakahara J., Shiina T., Suzuki N. (2017). Skeletal Muscle Involvement in Antisynthetase Syndrome. JAMA Neurol..

[43] Uruha A., Nishikawa A., Tsuburaya R.S., Hamanaka K., Kuwana M., Watanabe Y., Suzuki S., Suzuki N., Nishino I. (2017). Sarcoplasmic MxA expression: A valuable marker of dermatomyositis. Neurology.

[44] Zhang S.H., Zhao Y., Xie Q.B., Jiang Y., Wu Y.K., Yan B. (2019). Aberrant activation of type I interferon system may contribute to the pathogenesis of anti-MDA5 dermatomyositis. Br. J. Dermatol..

[45] Kim H., Gunter-Rahman F., McGrath J.A., Lee E., De Jesus A.A., Targoff I.N., Huang Y., O’Hanlon T.P., Tsai W.L., Gadina M. (2020). Expression of interferon-regulated genes in juvenile dermatomyositis versus Mendelian autoinflammatory interferonopathies. Arthritis Res..

[46] Monseau G., Landon-Cardinal O., Stenzel W., Schoindre Y., Mariampillai K., Barete S., Martel C., Masseau A., Meyer A., Terrier B. (2020). Systematic retrospective study of 64 patients with anti-Mi2 dermatomyositis: A classic skin rash with a necrotizing myositis and high risk of malignancy. J. Am. Acad. Dermatol..

[47] Concha J.S.S., Merola J.F., Fiorentino D., Werth V.P. (2018). Re-examining mechanic’s hands as a characteristic skin finding in dermatomyositis. J. Am. Acad. Dermatol..

[48] Katsumata Y., Ridgway W.M., Oriss T., Gu X., Chin D., Wu Y., Fertig N., Oury T., Vandersteen D., Clemens P. (2007). Species-specific immune responses generated by histidyl-tRNA synthetase immunization are associated with muscle and lung inflammation. J. Autoimmun..

[49] Okiyama N., Ichimura Y., Shobo M., Tanaka R., Kubota N., Saito A., Ishitsuka Y., Watanabe R., Fujisawa Y., Nakamura Y. (2021). Immune response to dermatomyositis-specific autoantigen, transcriptional intermediary factor 1gamma can result in experimental myositis. Ann. Rheum. Dis..

